# Symmetrization and Fock Space

**DOI:** 10.3390/e28090996

**Published:** 2026-09-06

**Authors:** Andreas Schlatter

**Affiliations:** The Quantum Institute, Gloversville, NY 12078, USA; schlatter.a@bluewin.ch

**Keywords:** symmetrized states, identical particles, Fock space, quantum statistics, entanglement

## Abstract

The assertion that quantum states of same-type particles have to be symmetrized because they are indistinguishable has led to a debate in the foundations of quantum mechanics around topics like “thisness” of particles and “surplus structure” in spaces of quantum states. We analyze the underlying mathematical notions and find that a central issue lies in the a priori identification of *n*-particle Fock space with the *n*-fold tensor product of single-particle spaces and the corresponding failure to recognize that multi-particle quantum states are ultimately the result of actions of Hamiltonians.

## 1. Introduction

In quantum mechanics, states of same-type particles are represented by symmetrized tensor products of single-particle spaces. The reason commonly indicated is that the particles, although being labeled, are indistinguishable, which is reflected in the permutation symmetry of the states. So, a two-particle electron state, for instance, would assume the typical form:

(1) $$\psi =\frac{1}{\sqrt{2}}\left(\left.\left|a\left(1\right)\right.\right\rangle ⨂\left|\left.b\left(2\right)\right\rangle \right.-\left|\left.b\left(1\right)\right\rangle \right.⨂\left|\left.a\left(2\right)\right\rangle \right.\right).$$

Equation (1) states that there is a particle, labeled one, and another particle, labeled two, which are in a superposition with their states a and b interchanged. The permutation (anti)symmetry of the labels reflects the fact that it is impossible to discern which particle is in which state. This idea fosters, of course, directly the debate about what “thisness” of a particle actually is [1,2,3,4,5,6,7,8,9]. In particular, it leads to three central questions. First, is it really indistinguishability which enforces symmetrization, and is Equation (1) with labeled particles in a twilight zone between states a and b the right representation? Secondly, what does one do with “unphysical” states like $\psi =\left.\left|a\left(1\right)\right.\right\rangle ⨂\left|\left.b\left(2\right)\right\rangle \right.$? And finally, are two electrons in a scattering experiment in an antisymmetric state, even if they initially are not entangled? The debate around these questions is particularly intricate if a position in space is added as a label in the states a,b, because our intuition strongly associates distinctiveness with position in space. Recently, it was rightly remarked [8] that the discussion has to broaden its scope away from debating the metaphysics of labeled tensor products towards a deeper look into the related physics, because states like in Equation (1) result in one way or another from interactions (even in phenomena like entanglement-swapping, there is always some pair of particles that has interacted). We are going to investigate these connections in a rigorous way and give answers to the questions formulated above. We do this by looking into the structure of the mathematical spaces in which quantum states are typically represented and start from two basic principles.

The first principle is the idea that particles are distinct only by their quantum numbers, i.e., they are “quanta”. This means that particles with the same quantum numbers are not distinct and can be aggregated without any individuation. It is like having 100 dollars or 50 euros in an account. The second principle is the quantum analogue of the empirical fact that two classical material systems are never simultaneously at the same position, namely Pauli’s exclusion principle, which says that two electrons can never simultaneously be in the same quantum state, i.e., share the same quantum numbers.

## 2. Fock Space

We are going to develop a well-known quantum theory with specific emphasis, however, on the three questions in the scope. We base the exposition in [7] (Chap. 3) to introduce the technical apparatus. The tool for introducing quanta is the notion of Fock space. Let there be a finite maximal set of commuting operators $A_{1},\ldots A_{K}$, producing (finitely many) eigenstates corresponding to arrays of quantum numbers $q_{j}=\left(q_{j}^{1},\ldots ,q_{j}^{K}\right),1\leq j\leq J.$ The numbering 1,…,J is conventional, but indices stay immutably attached to the respective eigenstates once determined. We now define a set of vectors $B=\left\{\left.\left.b\right|\;b=\left|n_{q_{1}},\ldots ,n_{q_{j}},\ldots ,n_{q_{J}}\right.\right\rangle ,n_{q_{j}}\epsilon N,1\leq j\leq J\right\}$, with their components signifying that $n_{q_{j}}$ particles are in the eigenstate associated with quantum numbers $q_{j}$. The total number of particles represented by a single vector in B is $\sum_{j=1}^{J}n_{q_{j}}=N,\;N\epsilon N$. To ease notation, we simply write $\left.b=\left|n_{1},\ldots ,n_{j},\ldots ,n_{J}\right.\right\rangle$. The vectors in B are orthonormal by definition:

(2) $$\left\langle m_{1},\ldots ,\right.\left.m_{j},\ldots ,m_{J}\left|n_{1},\ldots ,n_{j},\ldots ,n_{J}\right.\right\rangle =\prod_{j=1}^{J}\delta_{m_{j}n_{j}}.$$

They span a Hilbert space of variable, overall finite particle numbers (this means that $\underset{B}{\max}\sum_{j=1}^{L}n_{q_{j}}\leq L)$ $F=span\left(B\right)$, so-called Fock space, and are called the occupation-number basis. Bearing in mind the intuition behind the vectors in B and the fixed assignment of indices, it is clear that there is no physical distinction between basis vectors with merely permuted components. This just amounts to a permutation of notation without physical content. Therefore, the order of the numbering, 1≤⋯j≤⋯≤J, is fixed by convention. A special state in F is the vacuum state, $\left|\left.0\right\rangle \right.=\left.\left|0_{1},\ldots ,0_{j},\ldots {,0}_{J}\right.\right\rangle$, with no quanta at all (note that by (2) we have $\left\langle 0|0\right\rangle =1$).

Let $L\left(F\right)$ be the space of linear operators on F and let us define a set of linear operators ${\left\{{\hat{a}}_{j}\left(x_{j}\right)\right\}}_{1\leq j\leq J}\subset L\left(F\right)$ which transform one basis vector in B to another and which may also depend on position, $x_{j}\epsilon R^{3},$ next to the quantum numbers $q_{j}$. Space coordinates are added because these operators arise in practice from a second quantization, where solutions of differential equations are elevated to become operator-valued. We further work on a simultaneity slice in the non-relativistic regime. This eases the technicalities without loss of content. So, we define at a point $x_{j}\epsilon R^{3}$ the linear operators:

(3) $${\hat{a}}_{j}\left.\left(x_{j}\right)\left|n_{1},\ldots n_{j},\ldots ,n_{J}\right.\right\rangle =\sqrt{n_{j}}\;,\left.\left|n_{1},\ldots ,n_{j}-1,\ldots {,n}_{J}\right.\right\rangle ,\;\;\;n_{j}>0\;\;{\hat{a}}_{j}\left.\left(x_{j}\right)\left|n_{1},\ldots {,n}_{j},\ldots {,n}_{J}\right.\right\rangle =0,\;\;\;n_{j}=0,\;\;\;1\leq j\leq J.$$

By the inner product (2), there exists a set of adjoint operators ${\left\{{\hat{a}}_{j}^{+}\left(x_{j}\right)\right\}}_{1\leq j\leq J}\subset L\left(F\right)$ whose action on the basis vectors turns out to be, by the algebraic properties of F and by the definition of ${\left\{{\hat{a}}_{j}\left(x_{j}\right)\right\}}_{1\leq j\leq J}$, the following:

(4) $${\hat{a}}_{j}^{+}\left(x_{j}\right)\left.\left|n_{1},\ldots ,n_{j},\ldots {,n}_{J}\right.\right\rangle =\sqrt{n_{j}+1}\left.\left|n_{1},\ldots ,n_{j}+1,\ldots ,n_{J}\right.\right\rangle ,\;1\leq j\leq J.$$

In order to ease notation, we will in the sequel not explicitly state the dependency on $x_{j}\epsilon R^{3}$ and subsume the position under the quantum numbers $q_{j}$ and hence the index *j.* According to their action on Fock space, the two sets of operators are called annihilation and creation operators. The factors $\sqrt{n_{j}}\;,\sqrt{n_{j}+1}$ are chosen such that the composed operator ${\hat{N}}_{j}={\hat{a}}_{j}^{+}{\hat{a}}_{j}$ turns into the number operator:

(5) $${\hat{N}}_{j}\left.\left|n_{1},\ldots n_{j},\ldots ,n_{J}\right.\right\rangle =n_{j}\left.\left|n_{1},\ldots ,n_{j},\ldots ,n_{J}\right.\right\rangle ,\;\;\;1\leq j\leq J.$$

The composition of operators induces naturally a, generally non-commutative, relation on $L\left(F\right)$ by defining which operation is performed prior to the other. In contrast to permutations of components of the occupation-number basis in F, the relation in $L\left(F\right)$, given by operator composition, has a physical meaning in the simple sequencing of operations. One can now directly show by the orthonormality of the basis vectors $b=\left.\left|n_{1},\ldots n_{j},\ldots ,n_{J}\right.\right\rangle$ and by definitions (3) and (4) that there holds the following set of commutation rules (the commutator $is\;defined\;by\left[A,B\right]≔AB-BA$):

(6) $$\left[{\hat{a}}_{i}^{+},{\hat{a}}_{j}^{+}\right]=0,\;\;\;\left[{\hat{a}}_{i},{\hat{a}}_{j}\right]=0,\;\;\;\left[{\hat{a}}_{i},{\hat{a}}_{j}^{+}\right]=\delta_{ij},\;\;\;1\leq i,j\leq J.$$

In classical physics, it is an empirical fact that two material systems cannot simultaneously be at the same position. This fact translates to quantum physics by the Pauli principle for particles like electrons, which says that two electrons cannot simultaneously be in the same quantum state. Given the definitions (3) and (4) above, this has the immediate consequence that the occupation-number basis of electrons can only consist of vectors bϵB of the form:

(7) $$\left.b=\left|n_{1},\ldots ,n_{j},\ldots ,n_{J}\right.\right\rangle ,\;n_{j}=1\;or\;0,\;\;\;1\leq j\leq J.$$

Consequently, we immediately conclude for the sets of electron creation and annihilation operators, ${\left\{{\hat{b}}_{j}^{+}\left(x_{j}\right)\right\}}_{1\leq j\leq J}\subset L\left(F\right)$ and ${\left\{{\hat{b}}_{j}\left(x_{j}\right)\right\}}_{1\leq j\leq J}\subset L\left(F\right)$:

(8) $${\hat{b}}_{j}^{+}{\hat{b}}_{j}^{+}={\hat{b}}_{j}{\hat{b}}_{j}=0.$$

Because of Equation (8) and the fact that the operator $\frac{1}{\sqrt{2}}\left({\hat{b}}_{j}^{+}+{\hat{b}}_{i}^{+}\right)$ also produces a fermion, there also holds $\frac{1}{2}{\left({\hat{b}}_{j}^{+}+{\hat{b}}_{i}^{+}\right)}^{2}=0$, from which we derive the anticommutation rules (the anticommutator is defined $by\;\left\{A,B\right\}≔AB+BA\;$):

(9) $$\left\{{\hat{b}}_{j}^{+},{\hat{b}}_{i}^{+}\right\}=\left\{{\hat{b}}_{j}{,\hat{b}}_{i}\right\}=0.$$

Finally, by more algebra [7], there holds the full set of anticommutation rules:

(10) $$\;\left\{{\hat{b}}_{j}^{+},{\hat{b}}_{i}^{+}\right\}=0,\;\;\;\left\{{\hat{b}}_{j},{\hat{b}}_{i}\right\}=0,\;\;\;\left\{{\hat{b}}_{i},{\hat{b}}_{j}^{+}\right\}=\delta_{ij},\;\;\;1\leq i,j\leq J.\;\;$$

The spin-statistics theorem [10], for which a non-relativistic proof has been given in [11], says that if spin is among the quantum numbers $q_{j}$, then the commutation rules (6) lead to integer spin values, whereas the anticommutation rules (10) lead to half-integer spin values. Consequently, the theorem divides Fock space into two sectors, namely bosons and fermions with their respective creation and annihilation operators. Pauli’s principle holds, therefore, for all fermions. Note that both boson and fermion spaces can be generated recursively from the vacuum state by:

(11) $$\;\;\;\;\;\left.\left|n_{1},\ldots ,n_{j},\ldots ,n_{J}\right.\right\rangle =\frac{1}{\sqrt{n_{1}!}}\ldots \frac{1}{\sqrt{n_{j}!}}\ldots \frac{1}{\sqrt{n_{J}!}}{\left({\hat{a}}_{1}^{+}\right)}^{n_{1}}\ldots {\left({\hat{a}}_{j}^{+}\right)}^{n_{j}}\ldots {\left({\hat{a}}_{J}^{+}\right)}^{n_{J}}\left|\left.0\right\rangle \right.,1\leq j\leq J,$$

in the bosonic case and in the fermionic case by:

(12) $$\left.\left|1,\ldots ,1_{j},\ldots ,1_{J}\right.\right\rangle ={\hat{b}}_{1}^{+}\ldots {\hat{b}}_{j}^{+}\ldots {\hat{b}}_{J}^{+}\left|\left.0\right\rangle \right.,\;1\leq j\leq J.$$

This leads to the fermion annihilation operator:

(13) $${\hat{b}}_{j}\left|\left.\ldots ,1_{j},\ldots \right\rangle \right.=\pm {\hat{b}}_{j}{\hat{b}}_{j}^{+}\left|\left.\ldots ,0_{j},\ldots \right\rangle \right.=\pm \left(I-{\hat{b}}_{j}^{+}{\hat{b}}_{j}\right),\left|\left.\ldots ,0_{j},\ldots \right\rangle \right.=\pm \left|\left.\ldots ,\;0_{j},\ldots \right\rangle \right.,$$

with a $\left(+\right)$ sign, if the number of occupied states i<j is even and a $\left(-\right)$ sign, if it is odd.

We started the construction of Fock space from two principles. The first is the request that particles should be distinct by their quantum numbers only, and the second is the empirical fact that some particles, like electrons, cannot simultaneously be in the same quantum state. From there we derived a formal multi-particle Hilbert space F, called Fock space, with a particular basis B and sets of operators acting on B by increasing and decreasing the number of quanta. The composition of these operators shows specific permutation symmetries, which divide the elements of Fock space by recursion (11) and (12) into two sectors, bosons and fermions, characterized by integer and half-integer spin values, respectively. Note that there is no symmetrization of state vectors in the whole construction. Symmetry properties arise naturally at the level of the creation and annihilation operators, which are elements of the space of linear maps on F. These operators are also the building blocks of Hamiltonian operators. But how does the idea of permutation symmetry of quantum states factor into all this? That is what we treat next.

## 3. The Tensor Product

We have constructed Fock space F with basis vectors $\left.\left|n_{1},\ldots ,n_{j},\ldots {,n}_{J}\right.\right\rangle ,\;1\leq j\leq J,$ which indicate the occupation number of the eigenstates. It is possible to arrange Fock space in the following way as a direct sum:

(14) $$F={⊕}_{n=0}^{L}H^{n}.$$

Here $H^{n}\subset$ F represents the subspace with exactly n particles. Let us, for simplicity’s sake and without loss of generality, look at $H^{2}$. The occupation-number basis for $H^{2}$ consists of the bosonic sector of two types of vectors, namely $\left|\left.0_{1},\ldots ,1_{i},\ldots ,1_{j},\ldots {,0}_{J}\right\rangle \right.$ 1≤i<j≤J and $\left.\left|0_{1}\right.,\ldots ,2_{j},\ldots ,0_{J}\right\rangle$, 1≤j≤J, and in the fermionic sector of the first type only. An element of $H^{2}$ hence consists of two quanta, distinct by their quantum numbers, if at all, and by construction independent of each other. It is, however, an observed fact in quantum physics that two particles can have a type of inner correlation, called entanglement. This non-local correlation can be due to a shared and conserved quantum number stemming from interactions in the past. Such a correlation cannot be represented in Fock space, given the way F was defined as $F=span\left(B\right)$ in Paragraph 2, since there is no meaningful intra-mode relation defined on B and entanglement is not a property of a state alone but emerges when you specify how the state-space is framed, either by partitioning or by observing it through a restricted lens. To do this within F, an additional algebraic structure is needed. Therefore, one forms the tensor product of the single-particle spaces $H^{1}⨂H^{1}≔H^{2⊗}$, where the operation $\left(\cdot ⊗\cdot \right)$ defines the needed non-commutative relation (there holds v⊗w≠w⊗v, but there is an isomorphism v⊗w≅ w⊗v; there are alternative, more abstract possibilities to frame F and define entanglement). Note that $H^{2⊗}$ is neither for fermions nor for bosons identical to $H^{2}$. The crucial point is, however, that there are canonical isomorphisms from each sector $H_{B(F)}^{2}$ to subspaces of $H^{2⊗}$. Since $H_{B(F)}^{2}$ can be defined recursively (11, 12), the composition relation of operators in $L\left(F\right)$ and the tensor product in $H^{2⊗}$ must be compatible under such isomorphisms. In the case of bosons, the canonical isomorphism $H_{B}^{2}\to$ $H_{B}^{2⊗}$ could, in a first attempt, be the following map:

(15) $${\hat{a}}_{i}^{+}{\hat{a}}_{j}^{+}\left|\left.0\right\rangle \right.\to \left|\left.1_{i}\right\rangle ⨂\right.\left.\left|1_{j}\right.\right\rangle ,\;i\neq j,$$

where the order of indices in the tensor product mirrors one of the operators. But note the fact that ${\hat{a}}_{i}^{+}{\hat{a}}_{j}^{+}\left|\left.0\right\rangle \right.={\hat{a}}_{j}^{+}{\hat{a}}_{i}^{+}\left|\left.0\right\rangle \right.$ and (6) would demand that $\left|\left.1_{i}\right\rangle ⨂\right.\left|\left.1_{j}\right\rangle =\left|\left.1_{j}\right\rangle ⨂\right.\left|\left.1_{i}\right\rangle \right.\right.$, which is incorrect. Therefore, in order to reflect the symmetry of the operators in $H^{2⊗}$, we have to define for 1≤i,j≤J:

(16) $$I_{S}:\;\;{\hat{a}}_{i}^{+}{\hat{a}}_{j}^{+}\left|\left.0\right\rangle \right.\to \frac{1}{\sqrt{2}}\left|\left.1_{i}\right\rangle {⨂}_{S}\right.\left|\left.1_{j}\right\rangle ≔\right.\frac{1}{\sqrt{2}}\left(\left|\left.1_{i}\right\rangle ⨂\right.\left|\left.1_{j}\right\rangle \right.+\left|\left.1_{j}\right\rangle ⨂\right.\left|\left.1_{i}\right\rangle \right.\right),\;i\neq j,\;{\left({\hat{a}}_{i}\right)}^{2}\left|\left.0\right\rangle \right.\to \frac{1}{2}\left|\left.1_{i}\right\rangle {⨂}_{S}\right.\left|\left.1_{i}\right\rangle ≔\right.\left|\left.1_{i}\right\rangle ⨂\right.\left|\left.1_{i}\right\rangle \right..$$

Similarly, in the fermionic case, since ${\hat{b}}_{i}^{+}{\hat{b}}_{j}^{+}\left|\left.0\right\rangle \right.=-{\hat{b}}_{j}^{+}{\hat{b}}_{i}^{+}\left|\left.0\right\rangle \right.$ but $\left|\left.1_{i}\right\rangle ⨂\right.\left|\left.1_{j}\right\rangle \right.\neq -\left|\left.1_{j}\right\rangle ⨂\right.\left|\left.1_{i}\right\rangle \right.$, we have to define a map:

(17) $${I_{AS}\;\;:\hat{b}}_{i}^{+}{\hat{b}}_{j}^{+}\left|\left.0\right\rangle \right.\to \frac{1}{\sqrt{2}}\left|\left.1_{i}\right\rangle {⨂}_{AS}\right.\left|\left.1_{j}\right\rangle \right.:=\frac{1}{\sqrt{2}}\left(\left|\left.1_{i}\right\rangle ⨂\right.\left|\left.1_{j}\right\rangle \right.-\left|\left.1_{j}\right\rangle ⨂\right.\left|\left.1_{i}\right\rangle \right.\right),\;i\neq j.$$

These maps respect the operator symmetries at the level of state space, $H^{2⊗},$ and establish isomorphisms from the respective sectors of two-quanta Fock space to the symmetrized product of single-particle spaces:

(18) $$H_{B}^{2}≅H^{{2⨂}_{S}},{{\;H}_{F}^{2}\;≅H}^{{2⨂}_{AS}}.$$

## 4. Implications for the Three Questions

In the introduction, we posed three main questions around the symmetrization of quantum states to which we are now prepared to provide answers. Regarding the second question of surplus structure, the construction of the isomorphisms (18) shows immediately that there are no surplus elements, like the state $\left|\left.1_{i}\right\rangle ⨂\right.\left|\left.1_{j}\right\rangle \right.$, in a two-particle state space. Furthermore, as demonstrated in Paragraph 2, the distinction of quanta by the quantum numbers $q_{j}$ is sufficient to implement indistinguishability in Fock space $F=span\left(B\right)$. For the symmetrized states, $\left|\left.1_{i}\right\rangle {⨂}_{(A)S}\right.\left|\left.1_{j}\right\rangle \right.$, there is by construction no additional index available to identify the particles like the numbers 1,2 in Equation (1). This settles the first question in that symmetrization is not necessary to implement indistinguishability, and there are no ontological problems with “thisness” of particles, labeled by additional indices, since there are none available. The symmetrization in $H^{2⊗}$ is a result of the symmetries of creation and annihilation operators in $L\left(F\right).$ Ultimately, multi-particle occupation states are populated by the action of Hamiltonian operators, since Hamiltonians are built by creation and annihilation operators (note that free Hamiltonians just maintain the occupation numbers, while interactions, time-dependent external sources and the effects of non-stationary spacetime metrics increase or decrease them). It is explicitly shown in [8] how the *t*-channel and the *u*-channel in first-order electron–electron scattering produce an antisymmetric amplitude from a pair of incoming electrons. Indeed, since in the first-order amplitude of a two-particle scattering process there must appear a pair of creation operators acting on the vacuum state, due to normal ordering to the left of the annihilation operators, the isomorphisms $I_{A}$ and $I_{AS}$, (16,17), will apply and enforce symmetrization. So, the answer to the last question, namely whether two-particle states in a scattering experiment are always symmetrized, is a “yes” because the symmetries of the operators enforce it. If two electrons enter from t→−∞ with large spatial separation into a scattering event, however, the spatial overlap of their individual amplitudes can ab initio be zero, and the particles need not be entangled. Therefore, entanglement is not a necessary condition for symmetrization.

We learned that if we want to express the symmetries of creation and annihilation operators, being elements of $L\left(F\right)$, in an appropriate state space without referring to them, we need to add algebraic structure to Fock space in order to define a non-commutative relation. This is necessary because the occupation number basis has, as we introduced it in Paragraph 2, no meaningful intra-mode relation defined among its elements. The structure is found in the tensor product, and the symmetries of the operators are mapped to the symmetries of tensor products of single-particle states. From what we have seen, indistinguishability is not a sufficient condition to enforce symmetrization of multi-particle states, since indistinguishability is already achieved by $F=span\left(B\right)$. At the same time, entanglement is not a necessary condition for symmetrization. Symmetrized multi-particle states are populated by the actions of Hamiltonian operators and this important point is glossed over if Fock space is a priori defined via $F_{\left(F\right)B}={⊕}_{n=0}^{L}H^{n{⊗}_{\left(A\right)S}}$, as done in many textbooks.

Of course, we have not addressed the issue of the ontology of entities like electrons or photons per se and have tacitly used the admittedly vague term “particle” and from the beginning worked with “quanta” as outlined in [7]. It is well known that the particle-, the field-, and the quanta-interpretation of quantum field theory have their problems [9] (Chap. 6). Fock space only carries one of many unitary representations of free fields (i.e., the Poincaré group), and we know that it cannot be unitarily equivalent to an interaction representation by Haag’s theorem. In addition, the Reeh–Schlieder theorem tells us that the repeated action of operators, defined over a region $\Omega \subset M^{4}$, on the vacuum can produce any state, including one that is not localized in Ω (the vacuum state is a cyclic, separating state in the operator algebra ${\left\{A\left(\Omega \right)\right\}}_{\Omega \subset M^{4}}$). Over the years, there have been different answers to these challenges, not least the one that all these actions take place on entities, which are in a way propensities in modal space and which only occasionally, but under known conditions, appear in a definite state in our sensual world, e.g., in experiments [12]. It is probably fair to say that until today we do not know with certainty what the entities we are calling “electrons”, “photons”, etc. actually “are”. The more specific question about the reason for the existence and the meaning of symmetrized quantum states, however, can be answered by taking the physical meaning of mathematical structure as a beacon.

## Data Availability

No new data were created or analyzed in this study. Data sharing is not applicable to this article.
